# CRISPR/cas9, a novel genomic tool to knock down microRNA *in vitro* and *in vivo*

**DOI:** 10.1038/srep22312

**Published:** 2016-02-29

**Authors:** Hong Chang, Bin Yi, Ruixia Ma, Xiaoguo Zhang, Hongyou Zhao, Yaguang Xi

**Affiliations:** 1 Mitchell Cancer Institute, University of South Alabama, USA

## Abstract

MicroRNAs are small and non-coding RNA molecules with the master role in regulation of gene expression at post-transcriptional/translational levels. Many methods have been developed for microRNA loss-of-function study, such as antisense inhibitors and sponges; however, the robustness, specificity, and stability of these traditional strategies are not highly satisfied. CRISPR/cas9 system is emerging as a novel genome editing tool in biology/medicine research, but its indication in microRNA research has not been studied exclusively. In this study, we clone CRISPR/cas9 constructs with single-guide RNAs specifically targeting biogenesis processing sites of selected microRNAs; and we find that CRISPR/cas9 can robustly and specifically reduce the expression of these microRNAs up to 96%. CRISPR/cas9 also shows an exclusive benefit in control of crossing off-target effect on microRNAs in the same family or with highly conserved sequences. More significantly, for the first time, we demonstrate the long term stability of microRNA knockdown phenotype by CRISPR/cas9 in both *in vitro* and *in vivo* models.

MicroRNAs (miRNAs) are a set of short, non-coding, and small RNA molecules in length of 20–24 nt, which can generally repress gene expression at the post-transcriptional and/or translational levels by binding to the 3′ untranslated region (3′-UTR) of target genes^1^. Intriguingly, bioinformatics analyses based on the principle of “seed sequence matching” show that one miRNA can regulate hundreds to thousands of target genes; whereas one gene can be triggered by multiple miRNAs as well. Given the functional diversity of putative target genes, miRNAs are involved in almost all the cellular processes in both normal and pathological events. For example, miR-200 can target ZEBs, in turn, to upregulate E-cadherin to inhibit epithelial-mesenchymal transition (EMT)^2^; and repression of CDKN1A, E2F1 and PTEN by miR-17-92 cluster can result in uncontrolled cell proliferation^3^. Therefore, to understand the key signatures of miRNA in human diseases is critical to uncover complex pathogeneses and develop novel therapeutics.

Gain- and loss-of-function are the most frequently employed strategies to study a target gene in cell and molecular biology. Given the nature of miRNA that is the transcript from DNA, synthesized mimics or expression vectors to increase the abundance of miRNA in cells are broadly used in the most basic and translational researches. However, when compared to the effectiveness of overexpression strategies, the developed methodologies in downregulation of miRNA are relatively less robust^4^. This is partially due to the short length (~22 nt) of miRNA, which makes them more defensive towards the cleavage induced by other small molecules, such as siRNA.

As early as 1987, CRISPR (clustered regulatory interspaced short palindromic repeats) was first found in *Escherichia coli* K12. Over the past twenty years, these palindromic repeats have been identified in more than 40% bacteria and 90% archare^5^. CRISPR contains repeat sequences interspacing with spacers in terms of exogenous nucleotides from invading virus or plasmids, and its loci are often flanked by some associated endonucleases, such as cas. CRISPR is first transcribed to precursor CRISPR RNAs (pre-crRNAs) and then processed into crRNAs, which assemble with cas protein to form a complex that is able to trigger and cleave target DNA sequences^6^. However, CRISPR has not been well-known until 2013, when it emerged as a state-of-art genome editing tool, named as CRISPR/cas9 system^7^. It is composed of cas9 endonuclease cloned from *Streptococcus pyogenes* and a chimeric single-guide RNA (sgRNA) engineered from crRNA and a transactivating crRNA (tracrRNA); crRNA is responsible for recognizing and binding the sequences next to protospacer-adjacent motif (PAM), NGG, on the target DNA, whereas tracrRNA is essential to maintain cas9 nuclease activity^8^. Cas9 can cut the genomic DNA sequences to generate double strand DNA break (DSB), which can be patched by the repair system in a non-homologous end joining (NHEJ) manner with variable sizes of insertions or deletions (indels)^9^. Eventually, the expressions of target genes can be interrupted by the frame shift occurring in the coding regions^10^. Based on this theory, CRISPR/cas9 has been used to generate i*n vivo* mutation models in *C. elegant*, fly and mouse^9^,^11^,^12^. Furthermore, in a latest study, CRISPR/cas9 was reported to edit multiple genes including APC, SMADS4, TP53, KRAS and PIK3CA, in study of their contributions to colorectal cancer tumorigenesis^13^.

Given the pitfalls of current methodologies in miRNA silence versus the robustness of CRISPR/cas9 system in gene editing, we hypothesize that CRISPR/cas9 should be an innovative strategy in modulation of miRNA expression. In this study, by using human colon cancer lines, HCT116 and HT-29, as the research models, we transfect CRISPR/cas9 vectors targeting miR-17, miR-200c and miR-141 loci and examine alteration of these miRNA expressions accordingly. Aside from demonstrating that CRISPR/cas9 can robustly repress the expression of mature miRNAs companying with compellingly low off-target effect, we find that miRNA knockdown phenotypes caused by CRISPR/cas9 transient editing can be stably maintained in both *in vitro* and *in vivo* models for a long term (up to 30 days). Therefore, our results support that CRISPR/cas9 is a novel gene editing strategy with compelling robustness, specificity, and stability, in modification of miRNA expression.

## Results

### MiRNA knockdown by CRISPR/cas9

**Figure 1 f1:**
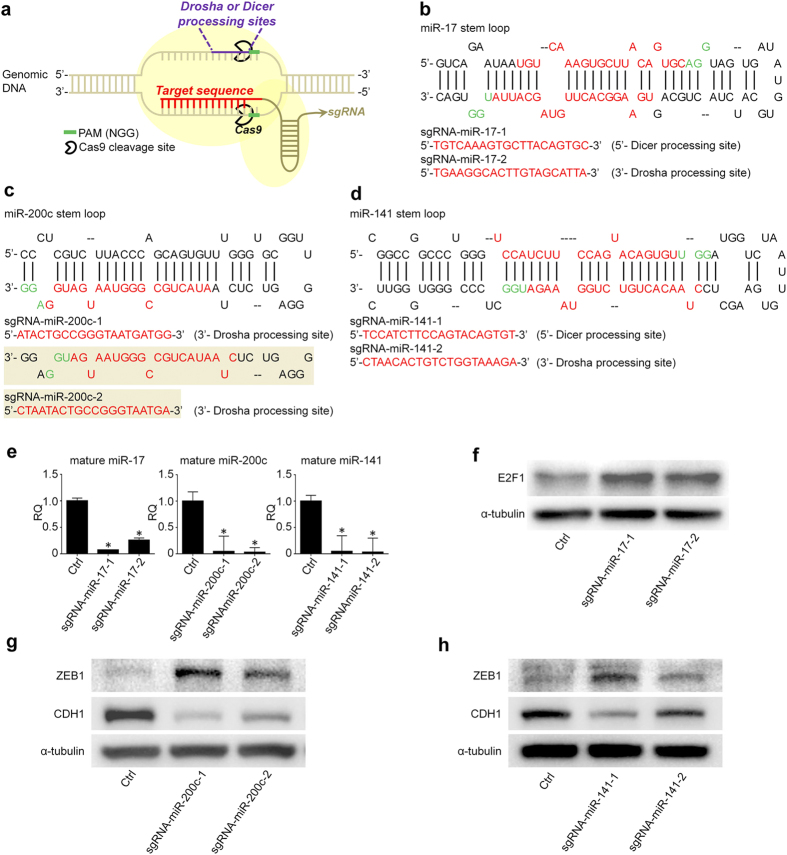
CRISPR/cas9 can significantly downregulate the expression of selected miRNAs. (**a**) Schematic diagram of CRISPR/cas9 system. (**b–d**) Design of sgRNAs for miR-17, miR-200c, and miR-141. Two sgRNAs are designed for each miRNA by using CRISPR DESIGN (http://crispr.mit.edu/). (**e**) CRISPR/cas9 with designated sgRNAs can significantly inhibit the expression of miR-17, miR-200c, and miR-141, respectively (n = 3). *P < 0.05. RQ: relative quantification. (**f**) sgRNA -miR-17 can upregulate the expression of the validated target of miR-17, E2F1. **(g,h)** sgRNA -miR-200c and sgRNA -miR-141 can upregulate the expression of the validated target of miR-200c and miR-141, ZEB1, which in turn represses the expression of E-cadherin. Ctrl is the CRISPR/cas9 vectors with sgRNA sequences targeting GFP. All the blots were run under the same experimental conditions and presented by using cropped images. Please refer to the supplementary for the full blots.

We aim to determine if CRISPR/cas9 targeting miRNA genomic DNA loci can robustly repress miRNA expression. As a result, we constructed CRISPR/cas9 vectors containing the individual sgRNAs with complementary sequences to miR-17, miR-200c, and miR-141 genes, respectively (Fig. 1a). Two sgRNAs were designed for each miRNA by using CRISPR DESIGN (http://crispr.mit.edu/), an online program that was developed and is maintained by Dr. Feng Zhang’s Lab at MIT. This tool can recommend sgRNA sequences for each input DNA fragment and analyze the potential off-target sites of individual sgRNA by bioinformatics blast with the whole genome DNA sequences^9^. In our study, two sgRNAs targeting the same miRNA were designed accordingly (Fig. 1b–d). Given the importance of Drosha and Dicer processing sites in process of miRNA biogenesis^14^, we triggered the PAMs (NGG) within/adjacent to these sites. The individual CRISPR/cas9 constructs were transiently transfected to HCT116 cells and the cells with CRISPR/cas9 were selected by puromycin treatment and expanded accordingly. As shown in Fig. 1e, the expression levels of mature miR-17, miR-200c and miR-141 declined up to 96% in the cells transfected with the designated CRISPR/cas9 constructs when compared to the control vectors, supporting the high efficiency of CRISPR/cas9 system in downregulation of mature miRNA expression.

The key signature of miRNA is the master role in repressing the expression of multiple genes at the post-transcription and/or translation levels. Thereby, we extended our observation to the expression of selected targets of these miRNAs. As shown in Fig. 1f, E2F1, one of the validated targets of miR-17^3^, appears to be induced in HCT116 cells transfected with CRISPR/cas9 against miR-17; knocking down miR-200c and miR-141 by CRISPR/cas9 can upregulate the expression of their co-target, ZEB1, which in turn enhances the repression of E-cadherin (Fig. 1g[Fig f1]h). The interaction between ZEB1 and E-cadherin has been exclusively studied in EMT of human cancer cells^15^. As expected, miR-200c or miR-141 knockdown by CRISPR/cas9 in HCT116 cells can also facilitate the cell invasion (Supplementary 2 Figure S2). Therefore, these results support that CRISPR/cas9 system can be used for miRNA loss-of-function studies.

### Indels generated by CRISPR/cas9 on miRNAs processing sites impede their biogenesis

**Figure 2:**
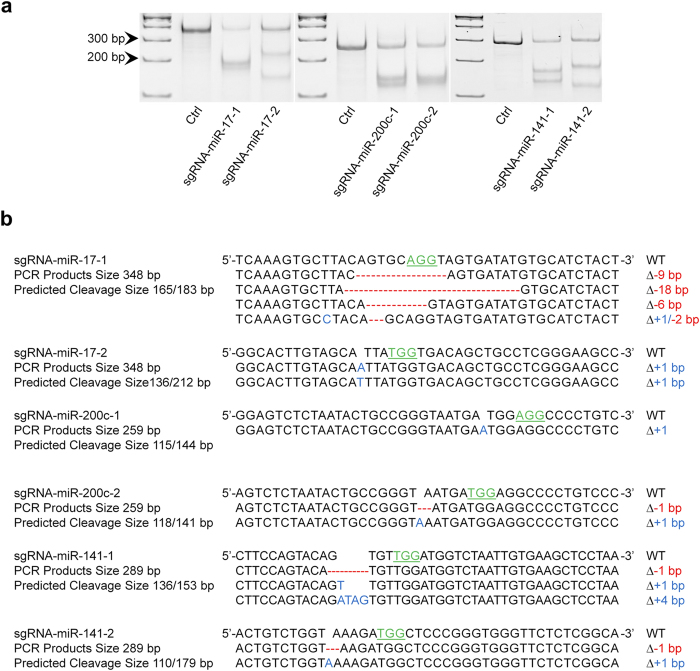
Detection and confirmation of indels generated by CRISPR/cas9 editing. (**a**) DNA cleavage by CRISPR/cas9 is detected by T7EN1 assay. (**b**) DNA sequencing confirms the deletions (Red) and insertions (Blue) generated by CRISPR/cas9 in selected miRNA genes. PAM sequences are highlighted by green. All the gels were run under the same experimental conditions and presented by using cropped images. Please refer to the supplementary for the full-length gel images.

As mentioned above, CRISPR/cas9 system can edit genome DNA sequences resulting in indels, which can be recognized by T7EN1 assay in an efficient manner^16^. After transfected CRISPR/cas9 vectors with individual sgRNA sequences targeting miR-17, miR-200c, and miR-141 to HCT116 cells, we detected the cleavages at target loci of these miRNAs by T7EN1 assay. As shown in Fig. 2a, the multiple site-specific bands were detected, which demonstrated the existence of indels. These results were further confirmed by DNA sequencing in which the random sizes of indels adjacent to PAM sequence were identified (Fig. 2b).

**Figure 3 f3:**
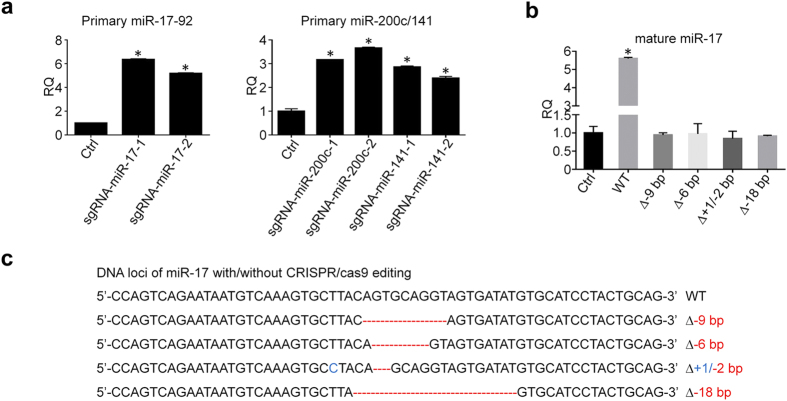
CRISPR/cas9 can impede the biogenesis process of miRNA. (**a**) CRISPR/cas9 induces the accumulation of primary miR-17-92 and miR-200c/141 clusters (n = 3). (**b**) Exogenous expression of miR-17 in HCT116 cells after transfected with wild-type miR-17 sequences and mutated sequences, respectively (n = 3). (**c**) DNA sequencing confirms the deletions (Red) and insertions (Blue) in the single clones with mutated miR-17 sequences. *P < 0.05. RQ: relative quantification.

Pri-miRNA is the direct transcript of miRNA gene and then processed by Drosha and Dicer to form short mature miRNA. Studies demonstrated that both flanking and internal structure of pri-miRNA can decide the efficiency of Drosha processing and, in turn, the biogenesis of miRNA^17^. As shown above, our results have shown that CRISPR/cas9 cleaves miRNA Drosha and Dicer processing sites leading to random sizes of indels on genomic DNA sequences. Then, we examined the primary miRNA expression. MiR-17 is one of six members in miR-17-92 cluster, and their primary miRNA is the gene transcript containing all 6 miRNA sequences. Likewise, miR-200c and miR-141 also share a same primary miRNA transcribed from miR-200c/141 cluster. As shown in Fig. 3a, pri-miR-17-92 and pri-miR-200c/141 clusters were upregulated, which imply that cleavage of miRNA Drosha and/or Dicer processing sites by CRISPR/cas9 could interrupt biogenesis of mature miRNA resulting in accumulation of pri-miRNAs. In order to further determine the effects of these indels on mature miRNA biogenesis, we cloned the wild-type miR-17 sequences as well as mutated miR-17 sequences with selected indels into pWPXL vectors, and then transfected them into HCT116 cells for exogenous expression of miR-17 in different single clones. As shown in Fig. 3b[Fig f3]c, mature miR-17 was significantly elevated in the cells transfected with wild-type miR-17 sequences, but not in the cells transfected with the single clones containing mutated miR-17 sequences. Moreover, not only single clones with large pieces of deletion (6–18 bp) can lead to the failure of mature miR-17 biogenesis, but also the clone with only 2 deletions and 1 insertion can impede the exogenous expression of miR-17 as well. These data strongly support that mutations generated by CRISPR/cas9 on the stem-loop structure of primary miRNA can result in the downregulation of mature miRNA by interrupting the process of biogenesis.

### Off-target effect of CRISPR/cas9 on miRNA editing

**Figure 4:**
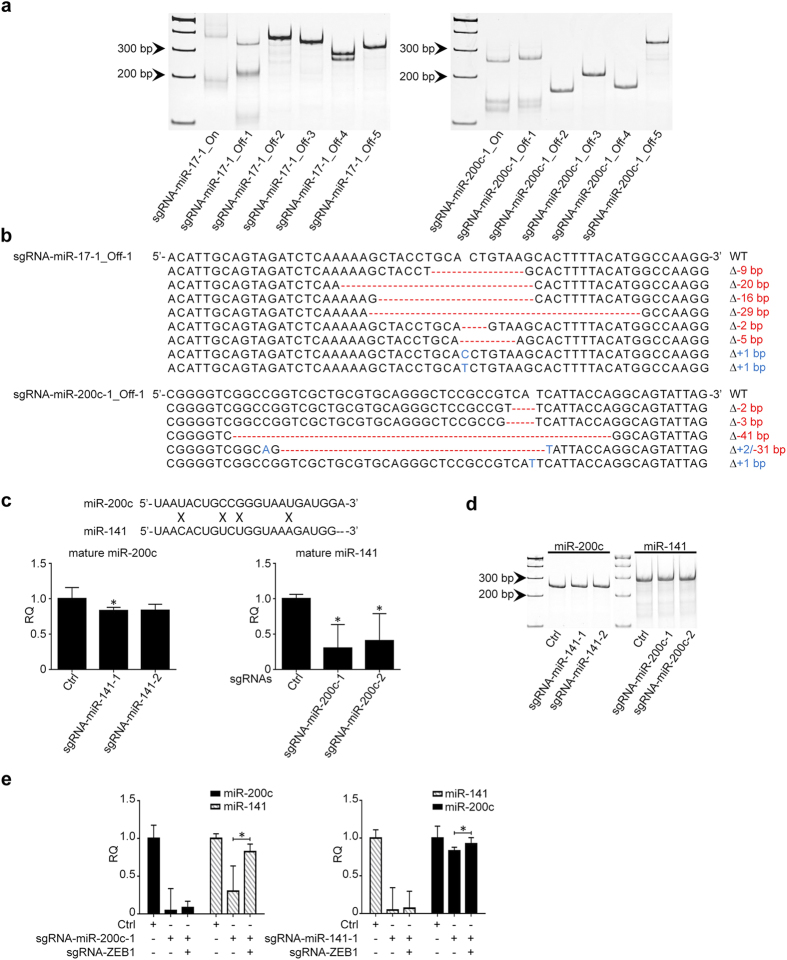
Determine the specificity of CRISPR/cas9 in editing miRNA. (**a**) CRISPR/cas9 cannot cleave the off-target sequences with > = 3 mismatches with sgRNA-miR-17-1 and sgRNA-miR-200c-1 by T7EN1 assay. “On” means on-target sequences; while “off” means off-target sequences. (**b**) DNA sequencing confirms the deletions (Red) and insertions (Blue) generated by CRISPR/cas9 in the off-target sequences containing < = 2 mismatches with sgRNA-miR-17-1 and sgRNA-miR-200c-1. (**c**) Crossing off-target effects between sgRNA-200c and sgRNA-141 as determined by qRT-PCR (n = 3). (d) T7EN1 assay confirms the DNA integrity of miR-200c subjected to sgRNA-miR-141 and that of miR-141 subjected to sgRNA-miR-200c. (**e**) Co-transfection of sgRNA-ZEB1 with sgRNA-miR-200c or sgRNA-miR-141 can significantly reduce crossing off-target effects on miR-141 or miR-200c, respectively (n = 3). *P < 0.05. RQ: relative quantification. All the gels were run under the same experimental conditions and presented by using cropped images. Please refer to the supplementary for the full-length gel images.

Off-target effect is the hallmark of all gene silence methodologies, including CRISPR/cas9 system. By using CRISPR DESIGN (http://crispr.mit.edu/), we predicted the off-targets of sgRNA-miR-17-1 and sgRNA-miR-200c-1 (Table 1), and examined their off-target effects accordingly. As shown in Fig. 4a, if the target sgRNA has more than 3 mismatches to the non-specific DNA sequences, there are no cleavages that can be detected by T7EN1 assay; however, if the mismatches are equal to or less than two, CRISPR/cas9 system can unbiasedly cleave the off-target sequences. The T7EN1 results were also confirmed by DNA sequencing (Fig. 4b). These results not only support the high specificity of CRISPR/cas9 system, but also provide a reference to guide the design of sgRNA sequences to reduce the off-target effects.

It was known that the traditional miRNA silence strategies, such as antisense inhibitors and sponge, are less robust when being used to inhibit the expression of miRNAs from the same family, given their highly conserved sequences with a few mismatches. For example, miR-200c and miR-141 are transcribed from the same genomic locus, and only have 4 mismatches in their mature sequences. Based on our results shown above, the CRISPR/cas9 targeting miR-200c should not influence the expression of miR-141 because the mismatches are more than 3. However, as shown in Fig. 4c, both sgRNAs-miR-200c generated cross expression inhibition on miR-141, vice versa. To address this unexpected result, we first examined DNA integrity of miR-200c and miR-141 genes in cells transfected with sgRNA-miR-141 and sgRNA-miR-200c by using T7EN1 assay, but only single bands were identified (Fig. 4d), which suggests that no cleavage was induced by CRISPR/cas9 and downregulation of miR-200c and miR-141 might not be caused by off-target effects. Given the regulatory loop between miR-200c/141 and ZEB1, we hypothesize that ZEB1 transcriptional regulation may be involved in these unexpected “off-target” phenotypes. It means when miR-200c is downregulated by CRISPR/cas9, ZEB1 will be inversely upregulated, which can in turn repress the expression of miR-141 at transcriptional level. Likewise, sgRNA-miR-141 can downregulate miR-200c via alternation of ZEB1 as well. To prove our hypothesis, we co-transfected CRISPR/cas9 constructs targeting ZEB1 and miR-200c or miR-141 into HCT116 cells, respectively. As shown in Fig. 4e, when ZEB1 was silent by CRSPR/cas9, the cross inhibitory phenotypes between miR-200c and miR-141 were nearly dismissed.

**Figure 5 f5:**
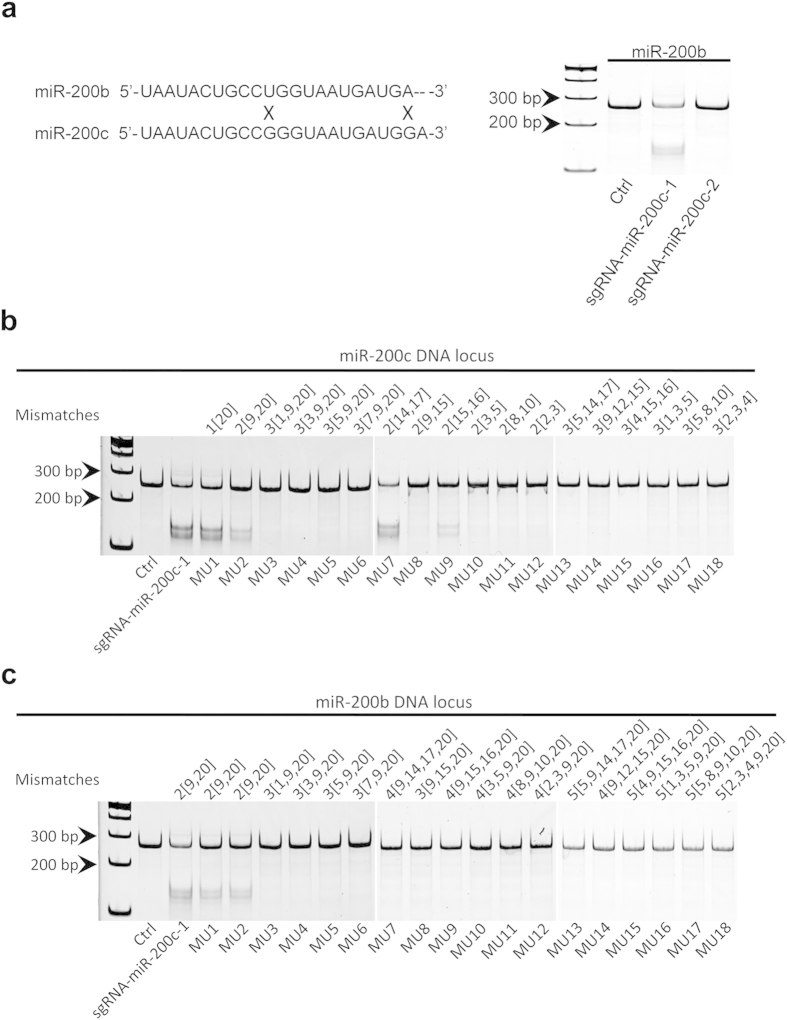
Crossing off-target effects of CRISPR/cas9 on miRNAs in the same family. (**a**) MiR-200b and miR-200c have only 2 different nucleotides in their mature sequences, and T7EN1 assay shows sgRNA-miR-200c-1 (with 2 mismatches) can cleave miR-200b DNA sequences, but not sgRNA-miR-200c-2 (without mismatches). We randomly made point mutations (1–4 bp) within the sequence of sgRNA-miR-200c-1, and examined their targeting effects on miR-200c (**b**) and potential off-target effects on miR-200b (**c**) by using T7EN1 assay. There are no cleavages if mutations (MU) are more than 3 bp. The digits in the figure can be interpreted by an example: 3[1,9,20] means there are 3 mutations locating at the 1^st^, 9^th^, and 20^th^ locus of sgRNA-miR-200c-1. All the gels were run under the same experimental conditions and presented by using cropped images. Please refer to the supplementary for the full-length gel images.

We also extend our observation to mature miR-200b that only has 2 different nucleotides from mature miR-200c. First, we blasted the sgRNA-miR-200c sequences to mature miR-200b sequence, and found that sgRNA-miR-200c-1 had 2 mismatches with miR-200b but sgRNA-miR-200c-2 did not align with miR-200b at all. Then, we performed the T7EN1 assay and found sgRNA-miR-200c-1 can edit the genomic DNA locus of miR-200b but not sgRNA-miR-200c-2 (Fig. 5a). These results suggest that the crossing off-target effects for the miRNAs at same family or with highly conserved sequences can be minimized by proper design of sgRNAs, which is very flexible and feasible because the design of sgRNAs can be anchored to dispersive PAMs that are easily identified in DNA sequences.

In order to further support our assumption, we randomly made point mutations (1–4 bp) within the sequence of sgRNA-miR-200c-1 (Table 2), and examined their targeting effects on miR-200c and potential off-target effect on miR-200b by T7EN1 assay (Fig. 5b[Fig f5]c). The results suggest that, if the numbers of mismatches are more than two, the mutated forms of sgRNA-miR-200c-1 show neither targeting effects on miR-200c, nor off-target effects on miR-200b, which suggest that high specificity and low off-target effect of CRISPR/cas9 in knockdown of miRNA. The T7EN1 results were also confirmed by DNA sequencing (data not shown).

### Stability of miRNA knockdown phenotypes by CRISPR/cas9

**Figure 6:**
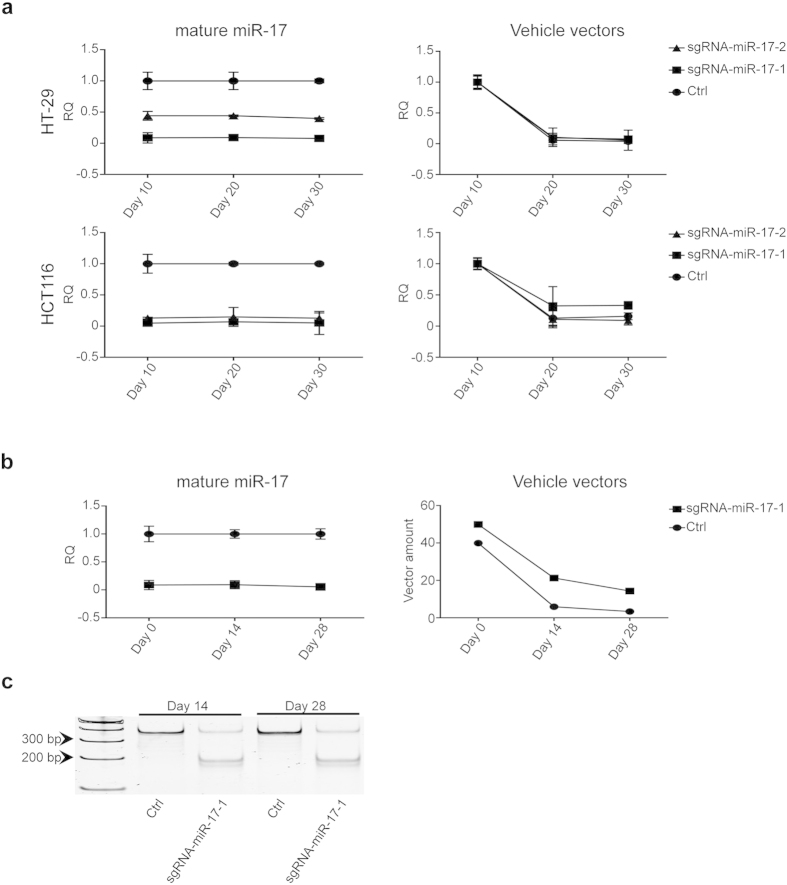
Stable miRNA knockdown phenotypes validated by *in vitro* and *in vivo* models. (**a**) MiR-17 knockdown phenotype can be maintained as long as 30 days in both HT-29 and HCT116 cells with transit transfection of CRISPR/cas9 constructs, although the vehicle vectors are being gradually lost (n = 3). (**b**) Xenograft tissues generated by subcutaneously injecting HT-29 cells with CRISPR/cas9 constructs targeting miR-17 in mice show stable knockdown phenotype versus gradually lost vehicle vectors up to 28 days (n = 3). (**c**) T7EN1 assay confirms the permeant cleavage of CRISPR/cas9 on miR-17 DNA sequences in xenograft tissues collected at Day14 and Day 28. RQ: relative quantification. All the gels were run under the same experimental conditions and presented by using cropped images. Please refer to the supplementary for the full-length gel images.

It was reported that CRISPR/cas9 could result in permanent cleavage on DNA sequence. However, to our knowledge, there is no published data showing if CRISPR/cas9 can establish stable phenotypes of gene knockdown for a long term in both *in vitro* and *in vivo* models after transfection of CRISPR/cas9. Therefore, we transiently transfected CRISPR/cas9 against miR-17 and the control vectors to HT-29 and HCT116 cells, respectively, and collected cells at Day 10, 20, and 30 after transfection. As shown in Fig. 6a, miR-17 is continuously downregulated in the cells with CRISPR/cas9 targeting miR-17, even though the amount of vehicle vectors is gradually dismissed over the course up to 30 days. To confirm the findings from our *in vitro* studies, we injected HT-29 cells with the miR-17 knockdown phenotype by CRISPR/cas9 to nude mice subcutaneously. We collected xenograft tumors at Day 14 and Day 28 for analysis, respectively. As shown in Fig. 6b, stable downregulation of miR-17 was validated in these xenograft tissues with gradual loss of the vehicle vectors. In addition, we checked the DNA cleavage of miR-17 in the xenograft tissues and found stable phenotypes in the samples collected in Day 14 and Day 28 (Fig. 6c). These results support that CRISPR/cas9 can generate stable miRNA knockdown phenotypes in both *in vitro* and in *vivo* models.

## Discussion

Robust, specific, and stable strategies for miRNA silence are essential for studying miRNAs functional capacity in human diseases^18^. The major loss-of-function technologies in miRNA studies include miRNA specific antisense inhibitors, miRNA sponges, and genetic knockout^19^. Antisense oligonucleotide inhibitors can bind with specific miRNAs reducing their repressive effect on target genes. Owing to the short length of miRNA, the binding affinity between miRNA and inhibitors is of concern when evaluating the efficiency of antisense oligonucleotide inhibitors. The innovative locked nucleic acid (LNA) technology appeared to address this pitfall and expand the application of antisense inhibitors in study of miRNA loss-of-function^20^. However, it is known that the degradation of miRNAs by antisense inhibitors rarely occurs given the short length. Therefore, even though losing the function, these mature miRNA sequences can still be detected by qRT-PCR, resulting in low or no correlations between the functional phenotype resulted from antisense inhibitors and the quantification of miRNA expression. This pitfall may cause difficulty in accurate measurement of antisense efficiency and potential toxicity. In addition, antisense inhibitors are limited to only short term studies because of irreproducibility after transfection. MiRNA sponges have tandem binding sites that can compete with specific targets dampening miRNA repressive function, which has shown the long term stability and improved efficiency in miRNA loss-of-function studies^21^. However, the miRNA sponge is vulnerable to Ago2-mediated endonucleolytic cleavage that limits its application in the selected models only. In addition, the expression efficiency of miRNA sponge constructs and the total number of binding sites contained in the sponge will decide the silence efficiency. Genetic knockout of miRNA is the most reliable technique on study of loss-of-function of miRNA with high efficiency and specificity for *in vivo* analysis; however, the manipulation procedure is complicated and the process is time consuming. Therefore, to establish a robust and convenient technique in study of miRNA loss-of-function is in need.

Recently, zinc-finger nucleases (ZFNs), transcription activator-like effector nucleases (TALENs), and CRISPR/cas9 system have been widely adapted for study of gene functions in variety of *in vitro* and *in vivo* models^22^,^23^. Compared to ZNF and TALEN, the design of CRISPR/cas9 is more convenient, flexible, and less costly, given the library of CRISPR/cas9 for human gene profile that has already been established^24^. In addition to a few publications that have reported the application of TALEN in editing or deletion of miRNA for establishing stable miRNA knockdown models^25^,^26^, the latest studies reported that CRISPR/cas9 could repress miRNA expression by targeting the terminal loop or 5′ region of pre-miRNA^27^,^28^. To be distinct from previous studies, we designed CRISPR/cas9 targeting the sequences within/adjacent to Drosha and Dicer processing sites in the secondary stem-loop structure of primary miRNAs, which are reported to be critical for processing miRNA biogenesis^14^. Our results show that the expression levels of mature miR-17, miR-200c, and miR-141 targeted by the designated CRISPR/cas9 constructs decrease up to 96%, respectively. It has been documented that inhibition of Drosha can lead to accumulation of primary miRNA (pri-miRNA) and in turn, reduction of mature miRNA biogenesis in cells^29^. As expected, we see the accumulation of pri-miRNA in the CRISPR/cas9 transfected cells. This phenotype was also observed when using CRISPR/cas9 to target 5′ region of pri-miRNA as well as using TALEN to interrupt miRNA biogenesis^28^,^30^, which suggests that the proper processing is of importance to maintain the function of miRNA. Innovatively, we extended our observations to the 3′ region of pri-miRNA, and we found that targeting both 3′ and 5′ regions can unbiasedly impede miRNA biogenesis process (Fig. 1). In addition, we cloned the individual mutations generated by CRISPR/cas9 and demonstrated their negative impact on processing mature miRNA. Therefore, our results support that CRISPR/cas9 is a robust strategy for miRNA loss-of-function studies when interrupting the biogenesis process.

We also observed the specificity of CRISPR/cas9, given the common concern on off-target effects for the gene silence strategies. Different studies have reported the high specificity of CIRSPR/cas9 system in various models^8^,^31^,^32^. For example, Cong *et al.* found that a single-nucleotide mutation in the seed sequence of CRISPR could completely abolish the cas9 activity, supporting the superior specificity of CRISPR/cas9 system^8^. However, a recent study reported that single or double mismatches could be tolerated by cas9 leading to high frequency off-target effect^33^, although the genome-scale sequencing results showed low incidence of off-target mutations in CRISPR/cas9 targeted human stem cells^34^. In our study, we evaluated the potential off-target effect of CRISPR/cas9 on the selected miRNAs. T7EN1 assay and DNA sequencing results showed that CRISPR/cas9 editing could unbiasedly occur if less than 2 mismatches are identified between the target sgRNA sequence and non-target DNA sequences; however, if there are 3 or more mismatches, no off-target effects were founded. Our results are highly consistent with the conclusion of a latest study on *Drosophila*^12^.

The highly conserved sequences in the same family miRNA raise a significant challenge for the application of the traditional miRNA silence strategies, such as antisense inhibitors and sponge. For example, the mature sequences of miR-200b and miR-200c have only 2 nucleotides difference, easily resulting in cross off-targeting effects. However, theoretically, CRISPR/cas9 is able to overcome this technical pitfall, given the flexibility in design of sgRNA targeting wide range of DNA sequences containing PAMs within the stem-loop structure of primary miRNA. In our study, we tested sgRNAs-miR-200c-1 and -2 for their off-target effects on miR-200b, and only found that sgRNA-miR-200c-1 containing 2 mismatches with miR-200b sequences could generate cleavage indeed. These results not only support the relatively high specificity of CRISPR/cas9 as we discussed above, but also indicate controllable crossing off-target effects among the miRNAs at the same family or with highly conserved sequences when using CRISPR/cas9 for miRNA loss-of-function studies.

Of note, we found that the CRISPR/cas9 constructs specifically targeting miR-200c could unexpectedly downregulate the expression of miR-141 as well. MiR-200c and miR-141 are two members of miR-200 family and their gene sequences are clustered in the same gene locus. Intriguingly, when using T7EN1 assay and DNA sequencing to examine the integrity of miR-141 gene, we failed to identify any non-specific cleavages caused by miR-200c CRISPR/cas9 constructs. This suggests that the downregulation of miR-141 might not be attributed to the off-target effect of CRISPR/cas9. After reading the related literatures, we learned that both miR-200c and miR-141 can target and repress the expression of ZEB1; whereas ZEB1 can inversely suppress miR-200c and miR-141 gene expression at the transcriptional level^2^. Therefore, when miR-200c is knocked down by CRISPR/cas9, the expression of ZEB1 can be elevated inversely, which in turn inhibits the expression of miR-141. To test our hypothesis, we co-transfected CRISPR/cas9 constructs targeting miR-200c and ZEB1 in HCT116 cells, respectively. As expected, miR-141 expression was rescued in the cells with co-transfection of CRISPR/cas9 constructs targeting miR-200c and ZEB1, which indicates that the downregulation of miR-141 was caused by ZEB1 transcriptional repression rather than off-target of CRISPR/cas9. These results again support the high specificity of CRISPR/cas9 on miRNA silence.

It was reported that the mutations caused by CRISPR/cas9 could be passed to the next generation in *C.elegant*^11^, and the mouse zygote with the indels would also develop normal blastocyst carrying the mutations^9^. These findings suggest that the indels generated by CRISPR/cas9 might not be possibly recovered in cells. It is known that the genome editing tools including CRISPR/cas9, ZFNs and TALENs can cause double-strand break (DSB), which can be repaired by either non-homologous end joining (NHEJ) or homology-directed repair (HDR), allowing these indels permanently remain in genomic contexts. To our knowledge, there are no studies that have provided direct evidence in support of long term stability of the cleavage of CRISPR/cas9 in miRNA gene locus in both *in vitro* and *in vivo* models. To test if the knockdown phenotype by CRISPR/cas9 can be stably maintained, we transiently transfected CRISPR/cas9 constructs targeting miR-17 into HCT116 and HT-29 cells, and then monitored the expression levels of mature miR-17 and vector sequences by using qRT-PCR, respectively. Our results show that the phenotype of miR-17 knockdown was maintained up to 30 days, while the vehicle vectors were lost gradually. We further validated our results by using animal models in which HT-29 cells transfected with CRISPR/cas9 constructs targeting miR-17 were injected subcutaneously. As expected, the *in vivo* results are highly consistent with the *in vitro* results, demonstrating that the indels from CRISPR/cas9 editing can be stably maintained in the cells, regardless of the existence of vectors carrying CRISPR/cas9. This is for the first time to evidence the long term stability of miRNA knockdown phenotype generated by CRISPR/cas9 in both *in vitro* and *in vivo* systems.

In this study, we investigated the potentials of CRISPR/cas9 as an innovative strategy serving miRNA loss-of-function studies, and our results support that CRISPR/cas9 is a more efficient, specific, economic, convenient, and stable technology for knocking down miRNA than the traditional methodologies including antisense inhibitors and sponge. The application of CRISPR/cas9 in miRNA editing will eventually strengthen our capability to study miRNA underlying functions in human diseases.

## Methods

### Cell culture

The human colon cancer HCT116 and HT-29 cells were purchased from American Type Culture Collection (ATCC). By following the manufactory’s instruction, HCT116 and HT-29 cells were cultured in McCoy’s 5A cell culture medium (Life Technologies) supplemented with 10% fetal bovine serum (Atlanta Biologicals). Cells were maintained at 37 °C in a humidified chamber supplemented with 5% CO_2_.

### CRISPR/cas9 to miRNAs construction and transfection

Plasmid lenti-CRISPR was requested from Dr. Feng Zhang’s lab through Addgene (#49535) and the plasmids constructed with protospacer sequence targeting GFP were used as the control vectors^24^. Protospacer sequences of CRISPR/cas9 against miR-17, miR-200c and miR-141 were designed by CRISPR DESIGN (http://crispr.mit.edu/)^24^. All specific target sequences were amplified and cloned into lenti-CRISPR vectors and verified by DNA sequencing. After the transient transfection of CRISPR/cas9 into cells by using Lipofectamine reagent (Life Technologies), puromycin (4 μg/mL) (Life Technologies) treatment for 24 hours was employed for selection and then cells were expanded in the regular culture medium. Primers sequences are listed in Supplementary.

### RNA isolation

TRIzol reagent (Life Technologies) was employed to isolate total RNAs from the cells and xenograft tissues. In brief, cells or gridded tissues were lysed in the mixture with 1 ml TRIzol reagent and 100 μl 1-bromo-3-chloropropane (BCP) (Molecular Research Center) by vigorously vortexing. After 10 min of centrifugation at 14,000 rpm at 4 °C, the upper aqueous phase was transferred to a new tube and an equal volume of isopropanol (Sigma-Aldrich) was added for precipitation. After washing with 75% ethanol for 15 sec, the pellets were air dried and eluted in nuclease-free water accordingly.

### SYBR-based qRT-PCR analysis

Specific stem-loop reverse transcription (RT) primers for mature miRNAs including miR-17, miR-200c and miR-141 were designed, respectively, and U6 was used as the endogenous control. As to primary miRNAs and GAPDH reverse transcription PCR, random hexamers supplied in the High-Capacity cDNA Reverse Transcription Kit (Applied Biosystems) were used. RT was performed at 37 °C for 2 h by incubating the mixture including 2 μg of total RNA, 2 μM RT primer for miRNAs or 1× random hexamers for primary miRNAs and GAPDH, 1× reverse transcription buffer and 1× dNTP at final concentration and nuclease-free water in a volume of 20 μl. For quantitative real-time PCR (qRT-PCR), 2× SYBR master mix (Roche) were utilized following the manufacture’s protocol. For mature miRNAs and primary miRNAs expressions analysis, the endogenous controls, U6 and GAPDH, were adapted, respectively. The relative expressions of target genes were estimated by 2-^ΔΔCT^ Method. The quantification of CRISPR/cas9 vectors in HCT116 and HT-29 cell lines was evaluated by SYBR-based qRT-PCR (n = 3). The yield of CRISPR/cas9 vectors in HT-29 xenograft tissues was measured by using an absolute quantification standard curve based on a dilution course of the control CRISPR/cas9 vectors.

### Western blotting

Anti-E-cadherin antibody was purchased from Cell Signaling Technology (24E10), anti-E2F1 (05–379) and anti-ZEB1 (ABE596) antibodies were bought from EMD Millipore. Anti-alpha-tubulin antibody (sc-8035) was got from Santa Cruz Biotechnologies. In brief, cell lyses were prepared by RIPA lysis buffer (Thermo Scientific) and the protein concentrations were determined by a BCA protein assay kit (Bio-Rad). Samples were carefully loaded to SDS–PAGE gel, separated and transferred to polyvinylidene fluoride membranes (PVDF) (Bio-Rad). The transblots were blocked by 5% non-fat milk then were incubated with primary antibodies at 4 °C overnight. After rinsing 3 times for 10 minutes each time with Tris-buffered saline solution containing 0.1% Tween 20 (TBST), peroxidase-linked secondary antibodies were incubated for 30 min. Finally, the transblots were incubated with SuperSignal West FemtoChemiluminescent Substrate (Thermo Scientific) for detection of HRP in dark chamber and images were captured by ChemiDoc™ XRS (Bio-Rad). The references for antibody dilution are alpha-tubulin: 1:1000; E-cadherin: 1:1000; E2F1: 1:1000; ZEB1: 1:250.

### T7EN1 analysis and DNA sequencing

The genomic DNA from the cells and xenograft tissues was extracted by using Wizard Genomic DNA Purification Kit (Promega). DNA fragments spanning target sites of CRISPR/cas9 were amplified by GoTaq DNA Polymerase (Promega) and PCR products were purified by GeneJET PCR Purification Kit (Thermo Scientific). T7EN1 assay is a known method to detect mismatched DNA^23^. In brief, total 200 ng of purified PCR products were denatured and reannealed in 1× NEBuffer 2 (NEB) in 20 μl volume in a thermocycler with following steps (95 °C, 5 min; 95-85 °C at −2 °C/s; 85-25 °C at −0.1 °C/s; hold at 4 °C)^35^. Then 10 U of T7EN1 enzymes (NEB, M0302L) were added to hybridized PCR products and reaction mix was incubated at 37 °C for 15 minutes. Reactions were stopped by addition of 2 μl 0.5 M EDTA. The products digested by T7EN1 were separated by 8% polyacrylamide gel electrophoresis (PAGE), stained with ethidium bromide (EB) and images were captured by ChemiDoc™ XRS (Bio-Rad). The PCR products subjected to T7EN1 enzyme digestion were also ligated to pGEM-T Easy vectors (Promega) for DNA sequencing.

### Plasmid construction and transfection

DNA fragments flanking pre-miR-17 were amplified by using DNA isolated from HCT116 cells transfected with sgRNAs targeting GFP and miR-17. The PCR products were qualitied by Agarose gel (Thermo Scientific) and purified by GeneJET PCR Purification Kit (Thermo Scientific), then ligated to pGEM-T Easy vectors (Promega) and sub-cloned into pWPXL vectors (Addgene, #12257). The sequences of the constructs were confirmed by DNA sequencing before transfection to the cells.

### *In vivo* validation

The protocol for use of animals was approved the Institutional Animal Care and Use Committee (IACUC) of University of South Alabama, and all the procedures were carried out in accordance with the approved guidelines. To determine the stability of miRNA knockdown phenotype by CRISPR/cas9, HT-29 cells (1 × 10^6^ cells) transfected with CRISPR/cas9 targeting GFP and miR-17 were mixed with 50% matrigel in a volume of 100 μl, respectively, and then injected into right flank of NCr-nu/nu mice (6–8 weeks old) subcutaneously. The xenograft tumors were dissected after 14 or 28 days incubation. Tissues were homogenized by using a small motor rotor and prepared for isolation of DNA and RNA.

### Statistical analysis

The data were analyzed by Fisher’s exact test depending on the Ct (cycle threshold) values included in qRT-PCR results. Differences were considered significant if the probability was less than 0.05.

## Additional Information

**How to cite this article**: Chang, H. *et al.* CRISPR/cas9, a novel genomic tool to knock down microRNA *in vitro* and *in vivo. Sci. Rep.* **6**, 22312; doi: 10.1038/srep22312 (2016).

## Supplementary Material

Supplementary Information

## Figures and Tables

**Table 1:** Off-target sequences of sgRNA-miR-17-1 and sgRNA-miR-200c-1 predicted by CRISPR DESIGN (http://crispr.mit.edu/).

SgRNAs		Sequence	PAM	Score	Mismatches	UCSC gene	Locus	Products Size (bp)	Predicted Cleavage Size (bp)
miR-17-1_	On	TGTCAAAGTGCTTACAGTGC	AGG			NR_029487	chr13:92002869	348	165/183
	Off-1	TGTAAAAGTGCTTACAGTGC	AGG	100	1	NR_029523	chrX:-133304272	316	110/206
	Off-2	TGTCTAAGCTCTTACAGTGC	TAG	1.4	3		chr3: + 151344524	357	106/251
	Off-3	GCTCAAAGTGCTGACAGTGC	TGG	1.2	3	NR_027774	chr17: + 43514416	340	99/241
	Off-4	TCTCAAAGAGATTACAGTGC	TAG	0.9	3		chr1:-183330797	275	88/187
	Off-5	TGTCATAGCTCTTACAGTGC	TAG	0.8	3		chr9: + 75156083	296	107/189
miR-200c-1_	On	ATACTGCCGGGTAATGATGG	AGG			NR_029779	chr12:7072907	259	118/141
	Off-1	ATACTGCCTGGTAATGATGA	CGG	2.4	2	NR_029639	chr1: + 1102540	270	133/137
	Off-2	AAAATGATGGGTAATGATGG	GAG	0.9	4		chr15:-45922506	156	52/104
	Off-3	ATAATTCCAGGTAATGATGG	AAG	0.9	3		chr9:-20452104	200	66/134
	Off-4	CTCCTGCTGGATAATGATGG	CAG	0.8	4		chr8: + 24105533	167	78/89
	Off-5	CTACTGCAGCATAATGATGG	AAG	0.7	4		chr18:-64345817	321	144/177

Note: “On” means on-target sequences, and “off” means off-target sequences. Mismatches are underlined.

**Table 2:** Alignment of mutated sgRNA-miR-200c-1sequences with miR-200c/b DNA loci.

	miR-200c DNA locus chr12: 7072907–7072926 ATACTGCCGGGTAATGATGG	Mismatches	T7EN1 cleavage	miR-200b DNA locus chr1: 1102542–1102561 ATACTGCCTGGTAATGATGA	Mismatches	T7EN1 cleavage
MU1	ATACTGCCGGGTAATGATGT	1 [20]	Yes	ATACTGCCGGGTAATGATGT	2 [9,20]	Yes
MU2	ATACTGCCAGGTAATGATGT	2 [9,20]	Yes	ATACTGCCAGGTAATGATGT	2 [9,20]	Yes
MU3	TTACTGCCAGGTAATGATGT	3 [1,9,20]	NO	TTACTGCCAGGTAATGATGT	3 [1,9,20]	NO
MU4	ATTCTGCCAGGTAATGATGT	3 [3,9,20]	NO	ATTCTGCCAGGTAATGATGT	3 [3,9,20]	NO
MU5	ATACAGCCAGGTAATGATGT	3 [5,9,20]	NO	ATACAGCCAGGTAATGATGT	3 [5,9,20]	NO
MU6	ATACTGTCAGGTAATGATGT	3 [7,9,20]	NO	ATACTGTCAGGTAATGATGT	3 [7,9,20]	NO
MU7	ATACTGCCGGGTACTGTTGG	2 [14,17]	NO	ATACTGCCGGGTACTGTTGG	4 [9,14,17,20]	NO
MU8	ATACTGCCCGGTAAAGATGG	2 [9,15]	Yes	ATACTGCCCGGTAAAGATGG	3 [9,15,20]	NO
MU9	ATACTGCCGGGTAAACATGG	2 [15,16]	NO	ATACTGCCGGGTAAACATGG	4 [9,15,16,20]	NO
MU10	ATTCAGCCGGGTAATGATGG	2 [3,5]	NO	ATTCAGCCGGGTAATGATGG	4 [3,5,9,20]	NO
MU11	ATACTGCGGCGTAATGATGG	2 [8,10]	NO	ATACTGCGGCGTAATGATGG	4 [8–10,20]	NO
MU12	AATCTGCCGGGTAATGATGG	2 [2,3]	Yes	AATCTGCCGGGTAATGATGG	4 [2,3,9,20]	NO
MU13	ATACAGCCGGGTACTGTTGG	3 [5,14,17]	NO	ATACAGCCGGGTACTGTTGG	5 [5,9,14,17,20]	NO
MU14	ATACTGCCCGGAAAAGATGG	3 [9,12,15]	NO	ATACTGCCCGGAAAAGATGG	4 [9,12,15,20]	NO
MU15	ATAGTGCCGGGTAAACATGG	3 [4,15,16]	NO	ATAGTGCCGGGTAAACATGG	5 [4,9,15,16,20]	NO
MU16	TTTCAGCCGGGTAATGATGG	3 [1,3,5]	NO	TTTCAGCCGGGTAATGATGG	5 [1,3,5,9,20]	NO
MU17	ATACAGCGGCGTAATGATGG	3 [5,8,10]	NO	ATACAGCGGCGTAATGATGG	5 [5,8–10,20]	NO
MU18	AATGTGCCGGGTAATGATGG	3 [2–4]	NO	AATGTGCCGGGTAATGATGG	5 [2–4,9,20]	NO

Note: Mismatches are underlined.
