# Supplementary material for: CRISPR/cas9, a novel genomic tool to knock down microRNA in vitro and in vivo
Source: Sci Rep. 2016 Feb 29;6:22312. doi: 10.1038/srep22312 (PMC4770416; doi:10.1038/srep22312)
Supplement: Supplementary Information [file srep22312-s1.pdf]

Supplementary 1:

Title: CRISPR/cas9, a novel genomic tool to knock down microRNA *in vitro* and *in vivo*

Authors: Hong Chang, Bin Yi, Ruixia Ma, Xiaoguo Zhang, Hongyou Zhao, Yaguang Xi

Primer Sequences

sgRNAs

sgRNA-miR-17-1: 5'-TGTCAAAGTGCTTACAGTGC-3'

sgRNA-miR-17-2: 5'-TGAAGGCACTTGTAGCATTA-3'

sgRNA-miR-200c-1: 5'-ATACTGCCGGGTAATGATGG-3'

sgRNA-miR-200c-2: 5'-CTAATACTGCCGGGTAATGA-3'

sgRNA-miR-141-1: 5'-TCCATCTTCCAGTACAGTGT-3'

sgRNA-miR-141-2: 5'- TAACACTGTCTGGTAAAGA-3'

sgRNA-GFP 5'-GAGCTGGACGGCGACGTAAA-3'

sgRNA-ZEB1 5'-ACCTGTCCATATTGTGATAG-3'

RT

miR-200c: 5'-GTCGTATCCAGTGCAGGGTCCGAGGTATTCGCACTGGATACGACTCCATC-3'

miR-141: 5'-GTCGTATCCAGTGCAGGGTCCGAGGTATTCGCACTGGATACGACCCATCT-3'

miR-17: 5'-GTCGTATCCAGTGCAGGGTCCGAGGTATTCGCACTGGATACGACCTACCT-3'

qRT-PCR

miR-200c-F: 5'-TCGGCGTAATACTGCCGGGTAATG-3'

miR-141-F: 5'- TAACACTGTCTGGTAAAG-3'

miR-17-F: 5'-CAAAGTGCTTACAGTGCA-3'

UNIVERSAL REVERSE: 5'-GTCGTATCCAGTGCAGGGTCCGAGGT-3'

GAPDH-F: 5'-TTGGCTACAGCAACAGGGTG-3'

GAPDH-R: 5'-GGTCTACATGGCAACTGTGAG-3'

CRISPR-UNI-F: 5’-CCATTAGGAGTAGCACCCACC-3’

CRISPR-UNI-R: 5’-GCTCCCAAGAACCCAAGGAA-3’

U6-F: 5’-CTCGCTTCGGCAGCACATATACT-3’

U6-R: 5’-ACGCTTCACGAATTTGCGTGTC-3’

miR-17 CLEAVAGE CLONING

miR-17-CLEV-PC1-F: 5’-GGATCCTCCCCATTAGGGATTATGCTGA-3’

miR-17-CLEV-PC1-R: 5’-GGATCCGCCAGAAGGAGCACTTAGGG-3’

T7EN1 ASSAY

miR-17-clev-F: 5’-TCCCCATTAGGGATTATGCTGA-3’

miR-17-clev-R: 5’-GCCAGAAGGAGCACTTAGGG-3’

miR-200c-clev-F: 5’-CCAGGGATCTGCAGCTTTTCC-3’

miR-200c-clev-R: 5’-CGCTCTCAGCTCAAGACGA-3’

miR-141-clev-F: 5’-TTGAGCTTGGGGTTGGCTC-3’

miR-141-clev-R: 5’-AGTGATTCGTCCCCCATCCA-3’

miR-17-OFF-1-F: 5’-TGCCAGAAGGGGCATTTAGG-3’

miR-17-OFF-1-R: 5’-CCAGGTGAGTCTGCATGGAT-3’

miR-17-OFF-2-F: 5’-GGAGTGAAGTACCTGGAGTCAC-3’

miR-17-OFF-2-R: 5’-CAGAAGCAAAGTGCAAACCCA-3’

miR-17-OFF-3-F: 5’-CCCTGTCTGTAGAGGGGTGA-3’

miR-17-OFF-3-R: 5’-ACTGCTGGGACAGGGTCTAT-3’

miR-17-OFF-4-F: 5’-CTGAGCACATGCCACCAAGA-3’

miR-17-OFF-4-R: 5’-GTGGCAATGGCAAATCCTAGT-3’

miR-17-OFF-5-F: 5’-TTTGTTTTGGATGTGGCCAGG-3’

miR-17-OFF-5-R: 5’-TGGCACCAACAAGTCTTAAGTT-3’

miR-200c-OFF-1-F: 5’-CTACTGAGCTTCCCAGCGAG-3’

miR-200c-OFF-1-R: 5’-TCACTCTCCCCAGAGCCATC-3’

miR-200c-OFF-2-F: 5’-TGACATGGGAGCATCCTGGG-3’

miR-200c-OFF-2-R: 5’-AAAGGCTCTCAGGGATTCACAG-3’

miR-200c-OFF-3-F: 5'-TCTACCACCCCTCTTGACCC-3'

miR-200c-OFF-3-R: 5'-GTGTTGTTCAGAGTTTTCACAGGA-3'

miR-200c-OFF-4-F: 5’-TATAAGCCAACCAGGGGCCA-3’

miR-200c-OFF-4-R: 5’-AGTGGCACAGAGAAGGAGGTA-3’

miR-200c-OFF-5-F: 5’-TGCCAAAAGGCAGTGCTAAAT-3’

miR-200c-OFF-5-R: 5’-AATCGTGCTTCTCATTGCCCT-3’

Supplementary 2:

Title: CRISPR/cas9, a novel genomic tool to knock down microRNA *in vitro* and *in vivo* Authors:

Hong Chang, Bin Yi, Ruixia Ma, Xiaoguo Zhang, Hongyou Zhao, Yaguang Xi

Figure S1: Full-length gels and blots.

Figure S2: The functional consequences of the microRNA knockdown by CRISPR/cas9.

Figure S1

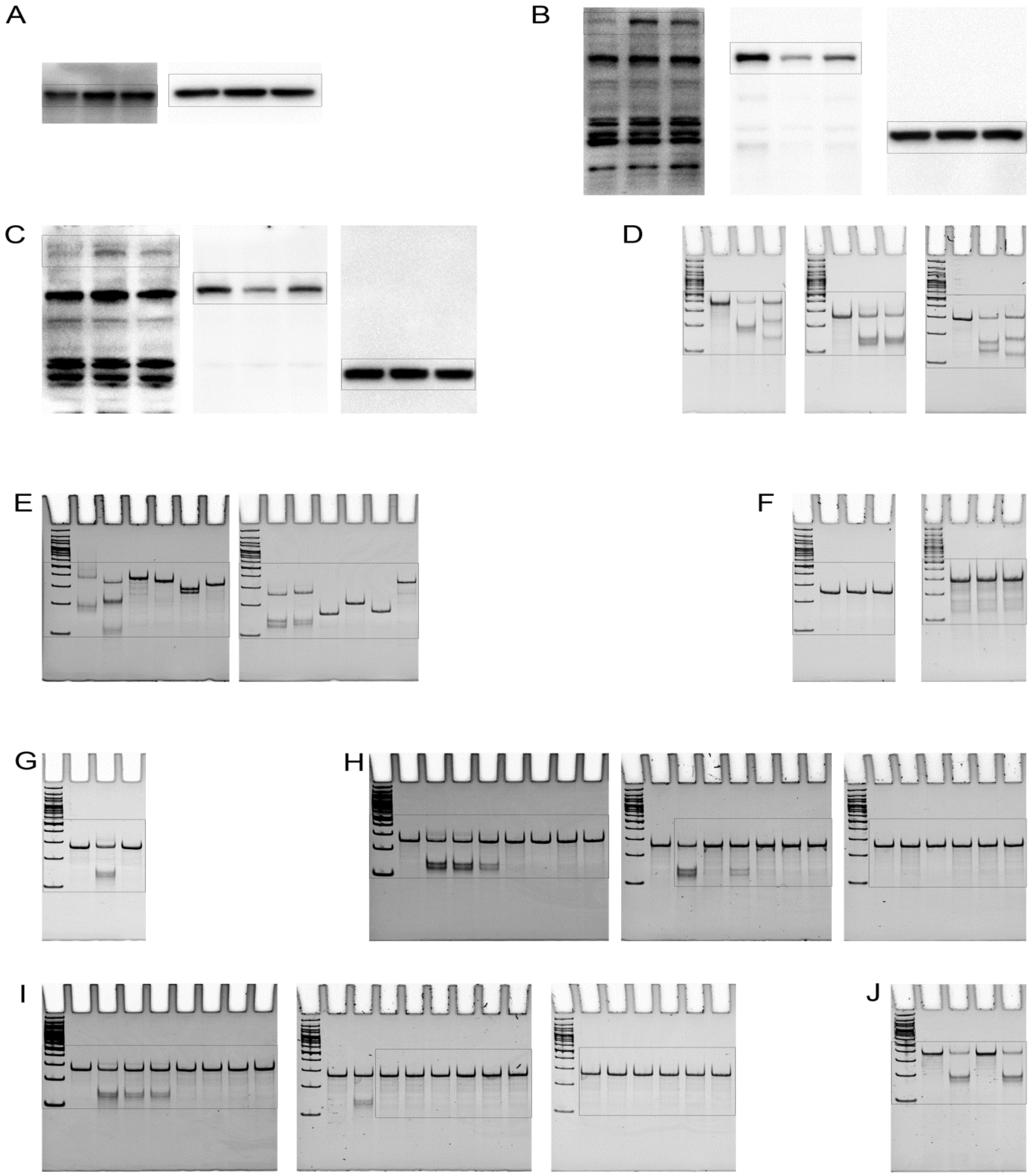

Figure S1. Full-length gels and blots. (A) Blot results of Figure 1f. (B) Full-length blot results of Figure 1g. (C) Full-length blot results of Figure 1h. (D) Full-length gel results of Figure 2a. (E) Full-length gel results of Figure 4a. (F) Full-length gel results of Figure 4d. (G) Full-length gel results of Figure 5a. (H) Full-length gel results of Figure 5b. (I) Full-length gel results of Figure 5c. (J) Full-length gel results of Figure 6c. Cropping is marked by black lines.

Figure S2

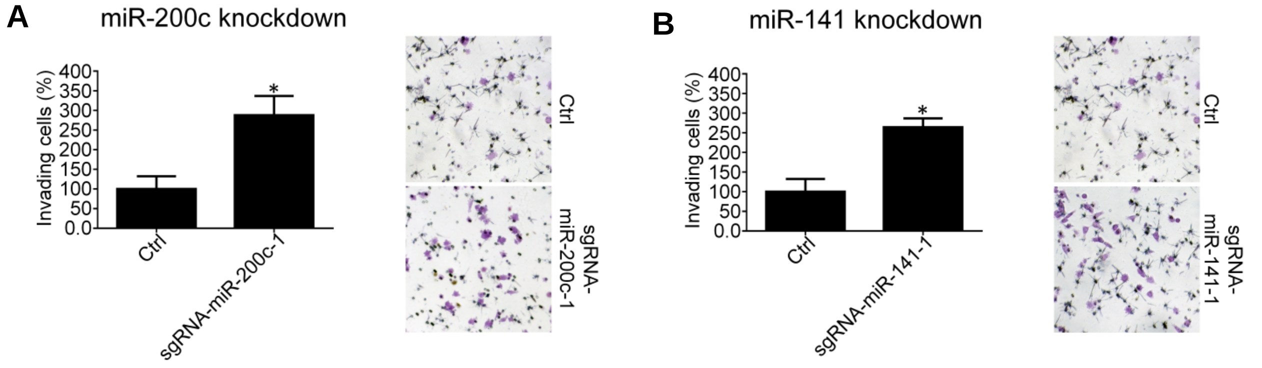

Figure S2: The functional consequences of the microRNA knockdown by CRISPR/cas9. CRISPR/cas9 constructs targeting miR-200c and miR-141 were transfected into human colon tumor HCT116 cells. BD Matrigel invasion assay was used to measure the invasive capability of HCT116 cells accordingly. As shown in (A) and (B), knocking down miR-200c and miR-141 can facilitate the invasion of HCT116 cells, respectively. *Left panel:* Quantification analysis of HCT116 cell invasion. *Right Panel:* The invading cells were fixed with formaldehyde and then stained with crystal violet. Five microscopic fields randomly chosen from each assay were counted individually, and the statistical significance between sgRNA and the vehicle control was determined by t-test (* $P<0.05$).
